# Transformer-Based Nonlinear Blind Source Separation for Anti-Jamming in DSSS Satellite Communications

**DOI:** 10.3390/s26072225

**Published:** 2026-04-03

**Authors:** Xiya Sun, Changqing Li, Jiong Li, Qi Su

**Affiliations:** 1 Graduate School, Space Engineering University, Beijing 101416, China; xiya_sun@hgd.edu.cn; 2 School of Space Information, Space Engineering University, Beijing 101416, China; lcqqcl5577@sohu.com (C.L.); suqi@hgd.edu.cn (Q.S.)

**Keywords:** nonlinear source separation, Transformer, satellite communication anti-jamming, interference suppression

## Abstract

High-power jamming may drive the radio-frequency (RF) front end of a satellite receiver into a nonlinear regime, thereby invalidating the linear superposition assumption underlying conventional excision and blanking methods. We formulate dual-receiver direct-sequence spread-spectrum (DSSS) anti-jamming as a nonlinear source-separation problem in complex baseband using stacked I/Q observations. We then propose a time-domain separator that jointly estimates the desired DSSS signal and the jammer on a designated reference receiver. The separator combines a multi-scale convolutional front end with a Transformer encoder and is pretrained on synthetic nonlinear mixtures that include multi-tone or burst jamming as well as typical satellite impairments, including Doppler/carrier-frequency offset (CFO), phase noise, multipath, and additive white Gaussian noise (AWGN). Robustness under high-jammer-to-signal-ratio (JSR) conditions is improved through high-JSR oversampling and JSR-aware loss reweighting. After Stage I supervised pretraining on labeled synthetic mixtures, an optional Stage II mixture-only adaptation step further refines the separator using nonlinear reconstruction consistency and lightweight communication-motivated priors. Across 1000 test mixtures with JSRs from −5 to 15 dB, SNRs from 15 to 25 dB, and cubic coefficients a∈[0,0.5], the proposed method improves the desired-signal scale-invariant signal-to-noise ratio (SI-SNR) from −4.79 dB for the mixture baseline to 13.32 dB after supervised pretraining and to 17.73 dB after mixture-only blind fine-tuning. Over the same test set, the failure rate (SI-SNR < 0 dB) decreases from 60.7% to 2.3%.

## 1. Introduction

Satellite communications and Global Navigation Satellite System (GNSS)-like DSSS links provide wide-area connectivity for navigation, broadcasting, emergency response, maritime and aviation services, and Internet of Things (IoT) backhaul [1]. Despite their broad deployment, such links are vulnerable to intentional jamming and unintentional co-channel interference because the wireless medium is open and the desired signal is typically received at very low power [2,3]. In practice, interference may be narrowband (e.g., multi-tone), wideband, intermittently bursty, or agile in time and frequency, and it can occur at a high jammer-to-signal ratio (JSR) [4,5]. Robust anti-jamming reception is therefore essential for maintaining link availability and service continuity under stringent power and spectral constraints [1].

Common countermeasures include direct-sequence spread-spectrum (DSSS) processing, adaptive notch filtering, time–frequency excision or blanking, and interference cancelation. When multiple antenna channels are available, spatial filtering and beamforming can further enhance suppression [2,4]. These techniques are most effective when the complex-baseband observation is well approximated by a linear superposition of the desired signal and interference in additive noise [2,6]. However, practical receivers may deviate from this assumption: high-power blockers can drive radio-frequency (RF) front-end components (e.g., low-noise amplifiers (LNAs), variable-gain amplifiers, limiters/clippers, mixers, and analog-to-digital converters (ADCs)) into nonlinear operation, producing spectral regrowth and intermodulation products [7,8,9]. Under strong jamming, nonlinear distortion can fold interference energy into the desired band and degrade acquisition, despreading, and detection even when the jammer would be separable under a linear model [8,9].

These observations motivate treating anti-jamming reception in complex baseband from the perspective of nonlinear blind source separation (BSS) [7]. We consider a multi-observation receiver in which the desired DSSS waveform and the jammer are jointly distorted by a memoryless nonlinear front end before additive noise is introduced. The separator takes stacked in-phase/quadrature (I/Q) observations from $N_{r}$ receivers as input, while supervised targets are defined on a designated reference receiver (see Section 3 for the formal model and notation). In the linear special case, established methods such as independent component analysis (ICA) and constant-modulus algorithm (CMA)-based equalization enable blind separation and have been widely studied [10,11,12]. In contrast, nonlinear mixing violates their underlying assumptions and can yield poor separation or unstable convergence, particularly under strong distortion and high JSR [7,13].

Nonlinear blind source separation (BSS) has been explored using post-nonlinear (PNL) models, kernelized independent component analysis (ICA), and related model-driven formulations [13,14]. While these approaches are theoretically well grounded, they often rely on restrictive identifiability conditions (e.g., invertible nonlinearities, sufficient excitation, or multiple observations) and can be sensitive to kernel design and hyperparameter tuning. Performance may further degrade in the presence of compound impairments that are common in satellite links, such as Doppler and carrier-frequency offset (CFO), phase noise, multipath, and time-varying interference [5,7]. Moreover, objectives that primarily optimize statistical independence may not directly translate into receiver-relevant waveform fidelity, which is critical for subsequent despreading and detection [15].

Recent advances in deep learning have enabled high-quality separation of long sequences using time-domain convolutional models, dual-path recurrent architectures, and Transformer-based separators [16,17,18,19]. In RF and communication settings, learning-based interference mitigation has also attracted attention [5,15,20,21]. However, many existing neural methods assume linear mixtures, rely on magnitude-only time–frequency representations that discard phase information, or require densely labeled clean references that are difficult to obtain in realistic satellite-reception scenarios [15,20]. In addition, models trained purely on synthetic data may suffer from domain shift in hardware deployment because nonlinearities, impairments, and channel effects are only approximated by simulation, which motivates source-free and test-time adaptation strategies [8,9,15,22].

Recent work has further expanded this landscape through deep anti-jamming methods for GNSS receivers [5], self-supervised nonlinear source separation [23], native complex-valued Transformer architectures for communication signals, and score-based RF source-separation models. These developments confirm the promise of learning-based interference mitigation, while also underscoring the need for domain-specific nonlinear separation frameworks that can operate under practical DSSS receiver impairments and limited access to clean reference signals.

To address these challenges, we propose a Transformer-based nonlinear source-separation framework for dual-receiver DSSS anti-jamming. The framework operates on stacked real-valued I/Q tensors derived from complex-baseband observations and jointly estimates the desired DSSS signal and the jammer on a designated reference receiver [16,17]. The novelty of the present work lies primarily in the nonlinear anti-jamming formulation, the two-stage learning strategy, and the robustness-oriented training design, rather than in claiming a fundamentally new generic Transformer backbone. Training follows a two-stage protocol. Stage I performs supervised pretraining on synthetic nonlinear mixtures that include multi-tone and burst jamming, Doppler/CFO, phase noise, multipath, and a memoryless cubic nonlinearity. Robustness under high JSR is further improved through high-JSR oversampling and JSR-aware loss weighting. Stage II performs mixture-only blind fine-tuning for domain adaptation by enforcing nonlinear reconstruction consistency and communication-motivated priors that encourage source separation while preserving waveform integrity [23,24]. We formulate inference as a nonlinear source-separation problem. Here, “blind” means that, during deployment and Stage II adaptation, the separator has access only to mixture observations and not to clean source references. Supervised learning is used only for Stage I pretraining on synthetic labeled data to obtain a robust initialization.

The main contributions of this work are summarized as follows:

- Nonlinear anti-jamming formulation: We formulate multi-observation DSSS anti-jamming under nonlinear RF front-end distortion as a nonlinear BSS problem in complex baseband, using stacked I/Q observations as input and applying supervision on a designated reference observation channel.
- Transformer-based separator for stacked I/Q representations: We develop a time-domain separator that processes stacked I/Q representations derived from complex-baseband observations and jointly estimates the desired signal and the jammer using a multi-scale convolutional front end and a Transformer encoder.
- Physics- and prior-informed training: We introduce a two-stage training protocol comprising supervised pretraining on synthetic nonlinear mixtures and mixture-only blind fine-tuning based on nonlinear reconstruction consistency and communication-motivated priors.
- High-JSR robustness mechanisms: We improve robustness under severe interference through high-JSR oversampling and JSR-aware per-sample loss reweighting during supervised pretraining.
- Diagnostic evaluation: We evaluate performance across ranges of SNR, JSR, and nonlinearity using separation metrics (e.g., SI-SNR) together with distributional diagnostics (e.g., percentiles and failure rates).

The remainder of this paper is organized as follows: Section 2 reviews related work on satellite and GNSS anti-jamming, blind source separation, and nonlinear mixing. Section 3 introduces the signal model and formulates nonlinear source separation in complex baseband. Section 4 presents the proposed separation framework and two-stage training protocol. Section 5 reports experimental setup, quantitative results, and discussion of key findings and limitations. Section 6 concludes the paper.

## 2. Related Work

### 2.1. Conventional Anti-Jamming in Satellite and GNSS Receivers

Satellite communication and GNSS receivers typically operate at very low received power levels, which makes the front-end observation highly susceptible to intentional jamming and unintentional co-channel interference. Existing countermeasures can be broadly grouped into (i) waveform-level resilience, (ii) spectral excision or adaptive filtering before despreading, and (iii) spatial filtering when multiple antennas are available. Although spread-spectrum modulation inherently suppresses narrowband interference (NBI), high-power or structured jammers can overwhelm the processing gain, necessitating additional front-end mitigation. A large body of work therefore studies excision filtering, adaptive filtering, and array-based processing for spread-spectrum interference rejection [2,4,6].

Adaptive notch and excision filters remain a standard choice for suppressing NBI prior to despreading. Open-loop or semi-blind designs estimate the jammer frequency and place a notch accordingly; however, overly aggressive excision may remove in-band signal energy and increase self-noise, degrading acquisition and tracking. Recent variants improve robustness by jointly tracking interference parameters (e.g., instantaneous frequency) and tuning multiple filter degrees of freedom to trade suppression against distortion (e.g., MPANF) [2,25].

For broadband, multi-tone, pulsed, or rapidly time-varying interference, mitigation is often performed in joint time–frequency representations or through adaptive logic that exploits sparsity across multiple domains, since the jammer may not be well localized in any single domain. Recent GNSS surveys emphasize that mitigation design should be driven by the threat model and that robustness to nonstationarity is a key requirement in practice [2,3].

When spatial diversity is available, space–time processing with antenna arrays is among the most effective defenses, particularly when the jammer arrives from directions distinct from the satellite line of sight. Space–time adaptive processing (STAP) jointly uses spatial and temporal degrees of freedom to form interference nulls while preserving the desired-signal response. Practical GNSS-oriented designs further account for carrier-phase integrity and navigation-signal distortion under coherent or closely spaced interferers [26].

Constraint-based formulations and beampattern synthesis have been proposed to reduce distortion and improve robustness relative to unconstrained STAP. For example, Xu et al. interpreted blind null steering as a two-dimensional notch generation problem and used frequency-invariant shaped-beam synthesis to achieve less distorted GNSS reception under multi-jammer conditions [27].

Summary: Conventional approaches are effective when their structural assumptions hold (e.g., jammer sparsity in time, frequency, or space and approximate linear superposition). Performance can deteriorate for overlapping or strongly nonstationary interferers, for limited snapshots, and especially when front-end nonlinearities invalidate linear models, which motivates blind source separation (BSS) and nonlinear BSS formulations [7,8,9,13].

### 2.2. Blind Source Separation for Interference Mitigation

Blind source separation (BSS), and in particular independent component analysis (ICA), provides a principled framework for recovering statistically independent sources from observed mixtures without labeled training targets. For linear instantaneous mixtures, ICA is supported by efficient algorithms (e.g., FastICA) and well-established independence criteria [11].

In communication and navigation receivers, BSS and ICA can act as a blind preprocessing stage for interference mitigation when multiple observations are available, for instance from antenna arrays, oversampling, or multi-band front ends. To better accommodate non-Gaussian interference and deviations from ideal linear mixing, recent studies also consider kernelized and nonlinear ICA and BSS variants [7,14].

To handle nonstationary or chirp-like jammers, many pipelines combine time–frequency analysis or adaptive filtering with subsequent separation stages, aiming to suppress interference while limiting distortion of the weak desired waveform [2,3,25].

Nevertheless, most classical BSS techniques assume approximate linearity, instantaneous mixing, and favorable source statistics (e.g., independence and non-Gaussianity). In satellite receivers, RF impairments and nonlinear components can substantially alter the effective mixture, motivating nonlinear BSS models and nonlinear-aware receiver processing [8,9,13].

### 2.3. Nonlinear Mixing and Nonlinear Blind Source Separation

Nonlinear distortion is common in satellite links, arising from receiver RF front ends and high-power amplification (e.g., TWTA and SSPA operation) as well as nonlinear coupling in practical hardware. Such effects motivate mixture models in which the observation is not a simple linear sum of sources [8,9]. A widely studied structure is the post-nonlinear (PNL) model, where a linear instantaneous mixture is followed by component-wise unknown but invertible nonlinearities [13].

General nonlinear mixing is not identifiable without additional assumptions; by contrast, the PNL class can be identifiable under meaningful conditions and has been addressed using mutual-information-based criteria and adaptive estimation schemes [14].

Recent work further parameterizes the inverse nonlinearities with flexible function approximators, including neural networks, while retaining a linear separation stage. This hybrid modeling reflects a broader trend toward learning-based inversion and compensation for receiver nonidealities [7,9,13].

From an RF impairment viewpoint, amplifier nonlinearities are often described with behavioral models such as polynomial or memory-polynomial representations. Although the appropriate model depends on the device and operating point, these formulations highlight that distortion can be structured and therefore amenable to data-driven compensation in an end-to-end design [8].

Implications for anti-jamming: When interference suppression is posed as separating the desired waveform from jammer components, front-end nonlinearities can transform an otherwise linear mixture into a nonlinear one. In this regime, linear BSS methods (e.g., ICA) may be insufficient, and nonlinear BSS, especially PNL-inspired structures, becomes directly relevant [7,8,13].

### 2.4. Deep Learning for Source Separation and Anti-Jamming

Deep learning has enabled high-quality source separation with end-to-end sequence models. In audio, time-domain encoder–separator–decoder architectures (e.g., Conv-TasNet) and dual-path recurrent networks (DPRNNs) showed that learned encoders coupled with sequence modeling can outperform classical time–frequency masking. Transformer-based separators (e.g., SepFormer) further improve long-context modeling via attention mechanisms [16,17,18,19].

Although developed for audio, the central idea is to learn a representation in which sources become separable. This idea also applies to communication mixtures, where signals exhibit structured modulation, coding, and synchronization. Communication-focused studies on data-driven blind synchronization and interference rejection indicate that leveraging domain structure and explicitly accounting for synchronization impairments can be critical to neural interference mitigation [20].

Within GNSS anti-jamming, learning-based methods have been used to complement array processing when classical snapshot or sparsity assumptions break down. For example, Xie et al. proposed an LSTM-based enhancement for GNSS array anti-jamming when the number of interferers challenges spatial sparsity, using an objective tailored to suppression performance and reporting improved cancellation in simulation [5].

A persistent practical limitation is the scarcity of labeled ‘clean’ targets for real satellite interference recordings, which motivates semi-supervised and self-supervised strategies that can adapt using mixture-only data [15].

In supervised multi-source separation, permutation-invariant training (PIT) addresses label ambiguity by optimizing over source permutations and has become a standard training component [28].

For unlabeled or weakly labeled data, consistency-regularization approaches such as Mean Teacher use an exponential moving average (EMA) teacher to provide stable targets for a student model, improving generalization under limited supervision [24]. Recent variants further refine the teacher–student formulation for challenging settings such as semantic segmentation [29].

Closer to nonlinear BSS, Webster and Lee introduced a fully self-supervised scheme based on multi-encoder autoencoders for nonlinear mixtures, where pathway specialization and reconstruction constraints encourage separable latent subspaces and enable source estimation via masking [23].

### 2.5. Summary and Research Gap

Overall, research on satellite and GNSS anti-jamming remains dominated by time- and frequency-domain excision and by array-based spatial nulling; when spatial diversity is available, STAP and constrained beampattern synthesis can improve robustness while reducing signal distortion [2,3,6,19,20].

However, many classical pipelines assume linear mixing and treat front-end nonlinearities as secondary impairments. Nonlinear BSS, particularly PNL models, provides a theoretical basis for separation under nonlinear distortion, yet optimization-based solutions can be fragile for long, high-dimensional sequences, nonstationary dynamics, and heterogeneous jammer types encountered in practice [7,8,9,13,14].

Deep separation models, especially Transformers, offer powerful long-context modeling and can learn representations that support separation under complex interference. Recent studies have highlighted three particularly relevant directions for this problem: deep anti-jamming for GNSS/satellite receivers [5], self-supervised nonlinear source separation with mixture-only adaptation [23], and native complex-valued neural architectures for communication signals [30,31,32]. Emerging score-based RF source-separation models further suggest that learned generative priors may become useful for communication-signal separation in the future [33,34]. Taken together, these studies indicate that the most promising path for satellite anti-jamming is not a direct transfer of generic source-separation backbones, but a domain-specific framework that couples nonlinear mixture modeling, label-efficient adaptation, and communication-aware inductive biases. In this context, the contribution of the present work is to develop such a framework for nonlinear DSSS anti-jamming under receiver front-end distortion, using stacked I/Q observations and a two-stage training strategy designed for high-JSR robustness and deployment-oriented adaptation. In this sense, the architectural choice serves the overall anti-jamming framework, rather than constituting the primary claimed novelty.

Modern broadband satellite communication systems, especially emerging LEO architectures, are considerably more complex than a basic single-link scenario, typically involving multibeam coverage, beam steering/beamforming, advanced modulation and coding, and hierarchical resource-management mechanisms [29]. These developments do not diminish the relevance of receiver-side anti-jamming; rather, they make robust per-beam or per-terminal reception even more important. In this context, the present work is intended not to model the entire satellite network architecture, but to address a complementary physical-layer subproblem: nonlinear interference mitigation when strong jammers or blockers drive the receiver front end into nonlinear operation. From this perspective, the proposed nonlinear separator can be viewed as a receiver-side front-end module that complements beamforming and conventional downstream synchronization, despreading/demodulation, and decoding in modern satellite receivers.

## 3. Problem Formulation and Signal Model

**Figure 1:**
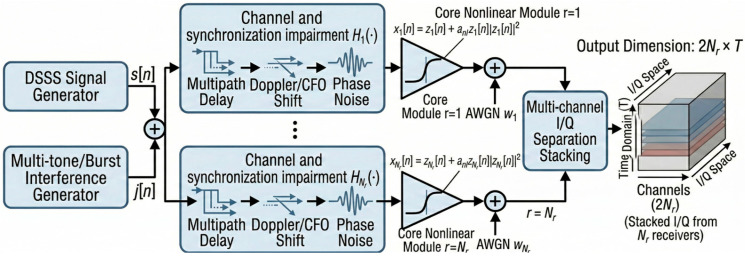
Synthetic dual-receiver mixture-generation pipeline.

This section presents the DSSS signal model, the jamming model, the channel and receiver impairments, and the memoryless cubic nonlinearity used to generate realistic complex-baseband mixtures. For receiver r, the corresponding complex-baseband observation is denoted by $y_{c}^{\left(r\right)}\in {\mathbb{C}}^{T}$. The separator does not operate directly on complex-valued tensors; instead, all receiver observations are converted into a stacked real-valued I/Q tensor $Y\in {\mathbb{R}}^{2N_{r}\times T}$, which is used as the network input. The synthetic dual-receiver mixture-generation pipeline is illustrated in Figure 1.

### 3.1. Notation and Data Representation

Let the discrete-time complex-baseband received sequence be $y_{c}[n]\in \mathbb{C}$, using chip-rate discrete-time samples with a fixed segment length T (default T=1024) and sampling rate $F_{s}$ (default $F_{s}=10\mathrm{MHz}$).

(1) $$y_{c}={\left[(y_{c}[0],y_{c}[1],\ldots ,y_{c}[T-1])\right]}^{T},y_{c}\in C^{T}.$$

To match real-valued neural-network inputs, we convert the complex multi-observation mixture into a $2N_{r}$-channel real I/Q tensor by stacking the real and imaginary parts of each receiver observation. We stack the channels in the order $\left[ℜ\{y^{\left(1\right)}\},ℑ\{y^{\left(1\right)}\},ℜ\{y^{\left(2\right)}\},ℑ\{y^{\left(2\right)}\},\ldots ,ℜ\{y^{\left(N_{r}\right)}\},ℑ\{y^{\left(N_{r}\right)}\}\right]$, consistent with the implementation. In this work, we use stacked real-valued I/Q tensors instead of a native complex-valued network implementation. This design choice is motivated primarily by implementation simplicity, optimization stability, and straightforward integration with the proposed multi-observation and mixture-only adaptation pipeline. Importantly, this representation does not mean that the real and imaginary parts are treated as independent signals. The I/Q channels are processed jointly across receivers, and the estimated I/Q pairs are reassembled into complex sequences before complex-domain supervision, blind reconstruction, and evaluation. Therefore, while the backbone is implemented in a real-valued form, the learning objectives remain explicitly tied to the complex-baseband structure of the communication signal.

We denote the number of observation receivers by $N_{r}$ (default $N_{r}=2$), and we use $r_{\mathrm{ref}}$ to denote the reference receiver index (default $r_{\mathrm{ref}}=0$) on which supervised targets and reported metrics are computed. Accordingly, the network input has dimension $2N_{r}\times T$, whereas each supervised target waveform has dimension 2×T on the reference receiver.

(2) $$Y=Q(y_{c})=\left[\begin{array}{c} ℜ\{y_{c}^{\left(1\right)}\}\\ ℑ\{y_{c}^{\left(1\right)}\}\\ ℜ\{y_{c}^{\left(2\right)}\}\\ ℑ\{y_{c}^{\left(2\right)}\}\\ \vdots \\ ℜ\{y_{c}^{\left(N_{r}\right)}\}\\ ℑ\{y_{c}^{\left(N_{r}\right)}\} \end{array}\right]\in {\mathbb{R}}^{2N_{r}\times T}$$

The separator outputs the desired-signal estimate $\hat{s}\in {\mathbb{R}}^{2\times T}$ and the jammer estimate $\hat{u}\in {\mathbb{R}}^{2\times T}$

(3) $$\hat{X}=\left[\begin{array}{c} \hat{s}\\ \hat{u} \end{array}\right]\in {\mathbb{R}}^{2K\times T},\;\;\;\;K=2.$$

Throughout this paper, u denotes the jammer component, whereas the imaginary unit is written as $j=\sqrt{-1}$. To avoid symbol conflicts, the jammer estimate is consistently denoted by $\hat{u}$, not $\hat{j}$.

### 3.2. DSSS Signal Model

Direct-sequence spread spectrum (DSSS) spreads each information symbol using a pseudo-noise (PN) chip sequence, providing processing gain and robustness against narrowband interference [30]. We generate a satellite-style baseband DSSS waveform using a symbol sequence a[k] and a per-symbol PN chip block c[k,i].

Let SF denote the spreading factor (chips per symbol). Symbols are mapped as

(4) BPSK:a[k]∈{+1,−1}.

(5) $$\mathrm{QPSK}:a[k]\in \left\{\frac{1}{\sqrt{2}}(\pm 1\pm j)\right\}.$$

Let the PN chips be

(6) c[k,i]∈{+1,−1},i=0,…,SF−1.

The chip-rate DSSS signal is

(7) s[n]=a[k]⋅c[k,i],n=k⋅SF+i.

**Figure 2:**
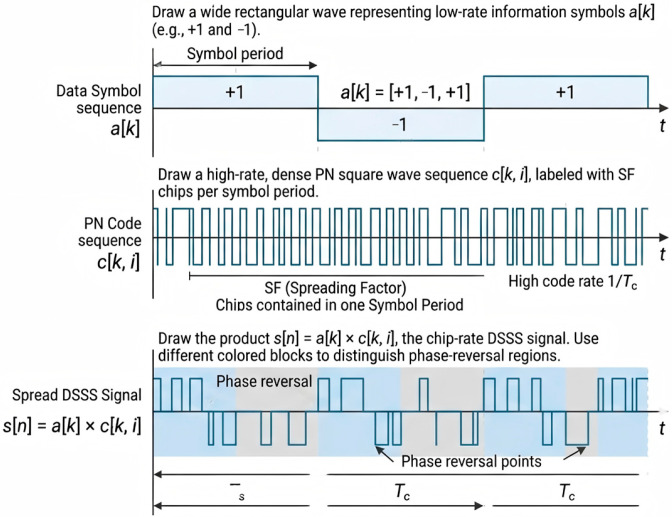
DSSS spreading process in the time domain.

With the default T=1024 and SF∈{16,32,64}, each segment contains an integer number of DSSS symbols, avoiding boundary ambiguity. The DSSS spreading process in the time domain is illustrated in Figure 2.

### 3.3. Jamming Model

We consider narrowband and multi-tone jamming as canonical threats in spread-spectrum receivers [2,3]. The complex-baseband jammer is modeled as a superposition of M tones with optional burst gating:

(8) $$u_{\mathrm{raw}}[n]=g[n]\frac{1}{\sqrt{M}}\sum_{m=1}^{M}A_{m}e^{j(2\pi f_{m}n/F_{s}+\phi_{m})}.$$

We denote the jammer by u[n]; the imaginary unit is written as $j=\sqrt{-1}$ to avoid ambiguity.

Burst gate definition (one simple realization): With probability $p_{b}$, draw a start index $n_{0}$ uniformly from the valid positions, and draw a duty cycle d∼Uniform[0.15,0.60]. Define

(9) $$g[n]=\left\{\begin{array}{c} 1,n_{0}\leq n<n_{0}+\left\lfloor dT\right\rfloor ,\\ 0,\mathrm{otherwise}, \end{array}\right.$$

and if no burst occurs, set g[n]≡1.

In our implementation, M∈{1,…,4}, $A_{m}=1$ for each tone, with overall normalization by $1/\sqrt{M}$, and $f_{m}∼\mathrm{Uniform}\left[-0.45\cdot \left(\frac{F_{s}}{2}\right),0.45\cdot \left(\frac{F_{s}}{2}\right)\right]$ to avoid edge effects near Nyquist.

JSR is defined on the reference receiver after the linear receiver/channel impairments in Section 3.4 and before the cubic nonlinearity and AWGN, so that the power ratio matches the receiver viewpoint used in evaluation.

For notational convenience, let ${\tilde{s}}_{r}[n]=H_{r}(s[n])$ and ${\tilde{u}}_{r}[n]=H_{r}(u[n])$, where $H_{r}(\cdot )$ denotes the receiver-specific linear impairment operator.

(10) $$P_{s}=\frac{1}{T}\sum_{n=0}^{T-1}{\left|{\tilde{s}}_{r_{\mathrm{ref}}}[n]\right|}^{2},\;P_{u}=\frac{1}{T}\sum_{n=0}^{T-1}{\left|{\tilde{u}}_{r_{\mathrm{ref}}}[n]\right|}^{2},$$

(11) $${\mathrm{JSR}}_{\mathrm{dB}}=10\log_{10}\left(\frac{P_{u}}{P_{s}}\right),$$

To match a target ${\mathrm{JSR}}_{\mathrm{dB}}$, we scale the jammer amplitude after burst gating:

(12) $$\beta =\sqrt{\frac{P_{s}10^{{\mathrm{JSR}}_{\mathrm{dB}}^{⋆}/10}}{P_{u}+\epsilon }},\;u[n]=\beta u_{\mathrm{raw}}[n].$$

where ε is a small constant for numerical stability. This yields per-segment power calibration (rather than only in expectation).

Training-set JSR oversampling: To improve robustness under high JSR, training samples may be drawn from a mixture distribution that oversamples 10–15 dB (e.g., with probability 0.6), while validation and test keep uniform sampling over [−5,15] dB.

### 3.4. Channel and Receiver Impairments

Satellite links can be approximated at baseband by Doppler and frequency offset, oscillator phase noise, multipath convolution, and additive noise. We optionally apply these impairments in synthesis.

Doppler and carrier-frequency offset (CFO):

(13) $$s_{d}[n]=s[n]\exp\left(j\left(2\pi \frac{f_{D}}{F_{s}}n+\phi_{0}\right)\right).$$

where $f_{D}$ is Doppler and CFO in Hz (default range ±2000 Hz), $F_{S}$ is the sampling rate, and $\phi_{0}$ is an initial phase.

Phase noise (discrete-time Wiener process):

(14) $$\phi [n]=\phi [n-1]+\Delta \phi [n],\Delta \phi [n]∼N\left(0,\sigma_{\phi }^{2}\right),$$

(15) $$s_{pn}[n]=s_{d}[n]\exp(j\phi [n]).$$

$\sigma_{\phi }$ is the per-sample random-walk increment standard deviation (default $3\times 10^{-4}$ rad).

Multipath (tapped-delay line, TDL):

(16) $$s_{c}[n]=\sum_{l=0}^{L-1}h_{l}s_{pn}[n-d_{l}],d_{l}\in \{0,1,\ldots ,D_{max}\}$$

where $h_{l}$ and $d_{l}$ denote the complex tap gain and integer delay of the l-th path, respectively.

### 3.5. Nonlinear Mixing Model

Practical RF front ends may exhibit nonlinear distortion, which couples signal and interference. We adopt a memoryless third-order polynomial approximation that captures dominant odd-order effects [8,9].

(17) $$z_{c}[n]=s[n]+u[n].$$

(18) $$x[n]=z[n]+a_{nl}z[n]|z[n]{|}^{2},a_{nl}\geq 0$$

The parameter $a_{nl}$ controls nonlinearity strength.

### 3.6. Additive Noise and SNR Definition

After nonlinear mixing and impairments, complex AWGN is added:

(19) $$y_{c}[n]=x[n]+v[n],v[n]∼CN(0,\sigma_{v}^{2})$$

We define SNR with respect to the desired-signal power Ps:

(20) $${\mathrm{SNR}}_{\mathrm{dB}}=10\log_{10}\left(\frac{P_{S}}{P_{V}}\right),P_{V}=\frac{1}{T}\sum_{n=0}^{T-1}|v[n]{|}^{2}$$

To match a target ${\mathrm{SNR}}_{\mathrm{dB}}$, we set

(21) $$P_{v}=\frac{P_{S}}{10^{{\mathrm{SNR}}_{\mathrm{dB}}/10}}$$

### 3.7. Problem Definition: Nonlinear Source Separation for Anti-Jamming

Combining the above factors, the complex-baseband observation can be written as

(22) $$z^{\left(r\right)}[n]=C^{\left(r\right)}(s[n])+C^{\left(r\right)}(u[n]),$$

(23) $$y_{c}^{\left(r\right)}[n]=z^{\left(r\right)}[n]+a_{nl}z^{\left(r\right)}[n]|z^{\left(r\right)}[n]{|}^{2}+v^{\left(r\right)}[n],\;\;\;\;r=1,\ldots ,N_{r}.$$

Here, $H_{r}(\cdot )$ denotes the receiver-specific linear impairment operator, including Doppler/CFO, oscillator phase noise, and multipath. In our simulator, both the desired signal and the jammer pass through the same receiver-dependent linear impairment chain before the memoryless cubic nonlinearity is applied. Finally, the multi-observation mixture is mapped to a real I/Q tensor $Y\in {\mathbb{R}}^{2N_{r}\times T}$ via channel-wise I/Q stacking, and supervised reference targets $(s,u)\in {\mathbb{R}}^{2\times T}$ are taken from a designated reference receiver $r_{\mathrm{ref}}$.

(24) $$y=Q(y_{c})\in {\mathbb{R}}^{2\times T}$$

The goal is to recover the desired DSSS signal (and optionally the jammer component) without explicit jammer priors, using a data-driven nonlinear source separation model [16,17]:

(25) $$(\hat{s},\hat{u})=f_{\theta }(Y),\;\;\;\;f_{\theta }:{\mathbb{R}}^{2N_{r}\times T}\to {\mathbb{R}}^{2K\times T}.$$

## 4. Proposed Method

### 4.1. Proposed Framework Overview

**Figure 3:**
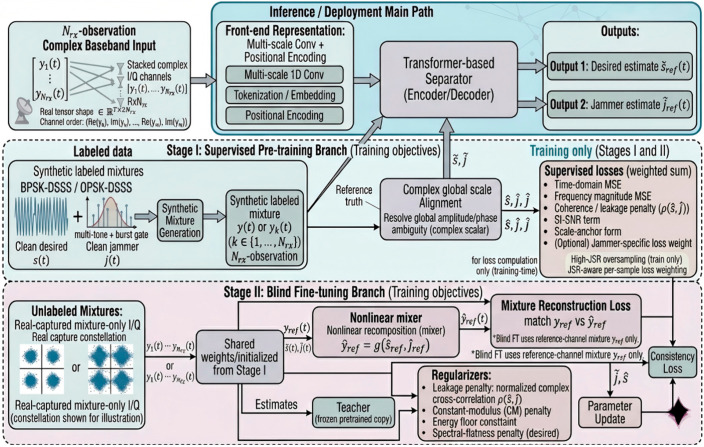
Overall inference and training pipeline of the proposed separator.

We first summarize the overall separation pipeline and the two-stage training strategy. Given stacked multi-observation I/Q mixtures, the separator estimates the desired DSSS component and the jammer on a designated reference channel. Stage I performs supervised pretraining on labeled synthetic nonlinear mixtures, while Stage II (optional) adapts the pretrained model to unlabeled mixtures via nonlinear mixture-reconstruction consistency and communication-motivated priors. The overall inference and two-stage training pipeline is shown in Figure 3.

The two stages serve distinct purposes rather than duplicating the same function. Stage I learns a robust nonlinear separation mapping from labeled synthetic mixtures and provides the initial model for the separator. Stage II is introduced to adapt the pretrained model to unlabeled target mixtures whose statistics may differ from those observed during synthetic pretraining. Stage II is therefore deployment-oriented. It addresses simulation-to-target-domain mismatch through mixture-only self-supervised refinement, which Stage I alone cannot provide because Stage I relies on labeled synthetic data and a fixed source distribution.

### 4.2. Model and Training Protocol

#### 4.2.1. Network Architecture

We use a time-domain separator that maps a stacked multi-observation I/Q tensor to estimates of the desired signal and the jammer on the reference channel. The network consists of a multi-scale convolutional front end, positional encoding, and a Transformer encoder backbone, with the main hyperparameters listed in Table 1.

**Figure 4:**
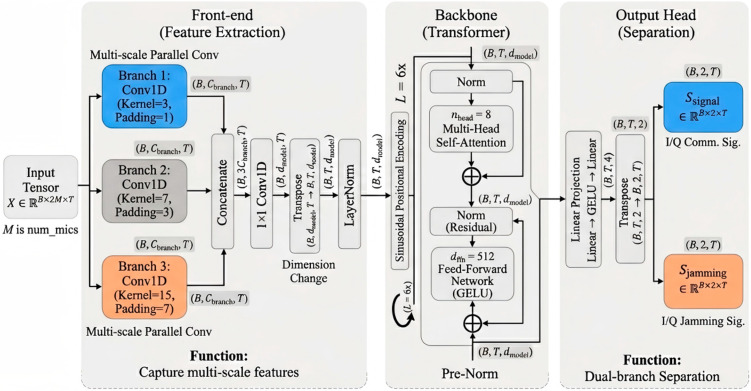
Architecture of the proposed multi-scale convolutional Transformer separator.

The current separator is implemented with a real-valued backbone operating on stacked I/Q channels rather than with native complex-valued Transformer layers. For the present study, this design provides a practical balance among model expressiveness, training stability, and implementation complexity, while remaining fully compatible with the proposed complex-domain alignment, complex-coherence regularization, and nonlinear mixture-reconstruction objectives. Extending the separator to a native complex-valued Transformer is an interesting direction for future work. The architecture of the proposed separator is shown in Figure 4.

#### 4.2.2. Supervised Pretraining on Synthetic Data

Supervised pretraining is performed on labeled synthetic mixtures with multi-observation inputs; supervised references are provided on a designated reference observation channel. Before computing waveform losses, the estimated and target I/Q pairs on the reference receiver are reassembled as complex sequences and aligned by a single complex scalar, which resolves the global complex scale/phase ambiguity.

(26) $$\alpha^{⋆}=\frac{\langle s_{c},{\hat{s}}_{c}\rangle }{\langle {\hat{s}}_{c},{\hat{s}}_{c}\rangle +\varepsilon },\;{\tilde{s}}_{c}=\alpha^{⋆}{\hat{s}}_{c}.$$

Here, $\langle a,b\rangle a=\sum_{n}a[n]b^{*}[n]$ denotes the Hermitian inner product.

Let $\langle \cdot ,\cdot \rangle$ denote the complex inner product. After global complex-scalar alignment, we minimize a weighted combination of time-domain MSE, frequency-domain magnitude MSE, and a complex-coherence penalty to encourage waveform fidelity, spectral agreement, and stable reconstruction.

(27) $$L_{\mathrm{time}}(s_{c},{\hat{s}}_{c})=\frac{1}{T}{\left\|{\tilde{s}}_{c}-s_{c}\right\|}_{2}^{2}.$$

(28) $$L_{\mathrm{freq}}(s_{c},{\hat{s}}_{c})=\frac{1}{T}{\left\|\left|\mathrm{FFT}({\tilde{s}}_{c})\right|-\left|\mathrm{FFT}(s_{c})\right|\right\|}_{2}^{2}.$$

(29) $$\begin{array}{l} \mathrm{coh}(\tilde{s},s)=\frac{\left|\langle \tilde{s},s\rangle \right|}{∥\tilde{s}{∥}_{2}∥s{∥}_{2}+\epsilon },\\ \mathrm{coh}(s_{c},{\hat{s}}_{c})=\frac{|\langle s_{c},{\tilde{s}}_{c}\rangle {|}^{2}}{\left(∥s_{c}{∥}_{2}^{2}+\varepsilon )(∥{\tilde{s}}_{c}{∥}_{2}^{2}+\varepsilon \right)}, \end{array}$$

(30) $$L_{\mathrm{coh}}(s_{c},{\hat{s}}_{c})=1-\mathrm{coh}(s_{c},{\hat{s}}_{c}).$$

We report a complex-valued variant of SI-SNR, defined after complex-scalar alignment, to evaluate I/Q waveform fidelity in the complex-baseband domain.

(31) $${\mathrm{proj}}_{s_{c}}({\tilde{s}}_{c})=\frac{\langle {\tilde{s}}_{c},s_{c}\rangle }{∥s_{c}{∥}_{2}^{2}+\varepsilon }s_{c},$$

(32) $$e={\tilde{s}}_{c}-{\mathrm{proj}}_{s_{c}}({\tilde{s}}_{c}),$$

(33) $$\mathrm{SI}-\mathrm{SNR}(s_{c},{\hat{s}}_{c})=10\log_{10}\frac{∥{\mathrm{proj}}_{s_{c}}({\tilde{s}}_{c}){∥}_{2}^{2}}{∥e{∥}_{2}^{2}+\varepsilon },$$

(34) $$L_{\mathrm{SI}}(s_{c},{\hat{s}}_{c})=-\mathrm{SI}-\mathrm{SNR}(s_{c},{\hat{s}}_{c}).$$

To avoid degenerate gain solutions permitted by scale invariance, we introduce a scale-anchor regularizer that penalizes log-domain gain drift by minimizing ${\left(\log(|\alpha |+\epsilon )\right)}^{2}$, which yields a symmetric penalty for both gain inflation and attenuation.

(35) $$L_{\mathrm{scale}}={\left(\log(|\hat{\alpha }|+\varepsilon )\right)}^{2}$$

The supervised objective for each source is defined as a weighted sum of the component losses described above.

(36) $$L_{\sup}^{\left(s\right)}=\lambda_{t}L_{\mathrm{time}}+\lambda_{f}L_{\mathrm{freq}}+\lambda_{c}L_{\mathrm{coh}}+\lambda_{si}L_{\mathrm{SI}}+\lambda_{sc}L_{\mathrm{scale}}.$$

The loss terms in Equation (36) play complementary roles rather than serving the same function. Specifically, $L_{\mathrm{time}}$ enforces pointwise waveform fidelity after complex-scalar alignment, while $L_{\mathrm{SI}}$ directly optimizes scale-invariant reconstruction quality in the complex-baseband domain. $L_{\mathrm{freq}}$ promotes spectral agreement between the estimate and the reference, which helps preserve the spectral structure of the separated waveform under strong interference. $L_{\mathrm{coh}}$ encourages phase-consistent reconstruction by increasing the normalized complex correlation between the estimate and the target. Finally, $L_{\mathrm{scale}}$ serves as a stabilizing regularizer that suppresses degenerate gain drift permitted by scale-invariant objectives. Taken together, these terms allow the supervised objective to balance waveform fidelity, spectral and correlation consistency, and optimization stability. This design is better suited to downstream communication processing than relying on a single reconstruction criterion alone.

In practice, the loss weights were chosen empirically so that the optimization remains dominated by desired-signal reconstruction quality, while auxiliary terms provide spectral/correlation consistency and training stability without overwhelming the primary objective. Accordingly, the principal fidelity terms were assigned the dominant contribution, whereas the scale-anchor and jammer-supervision terms were kept small. In the implementation used in this study, the default supervised-pretraining weights were set to $\lambda_{t}=1.5$, $\lambda_{f}=0.5$, $\lambda_{c}=0.3$, $\lambda_{\mathrm{si}}=2.0$ and $\lambda_{\mathrm{sc}}=0.02$, with a jammer-supervision weight of 0.1. In addition, JSR-aware weighting was applied only to the desired-signal loss, beginning at 5 dB JSR with linear growth and a capped multiplier, so that severe interference cases receive greater emphasis without destabilizing training. The final settings were selected according to validation performance together with reliability-oriented diagnostics such as failure rate and lower-tail behavior.

When both sources are available, losses are computed for the desired component and (optionally) for the jammer component; the two terms are then combined using a jamming-supervision weight to control the degree of jammer supervision.

To emphasize worst-case interference conditions, we optionally apply JSR-aware reweighting to the desired-component loss so that higher-JSR samples contribute more strongly during optimization. The key settings and default loss weights for supervised pretraining are summarized in Table 2.

Details of the optional mixture-only blind fine-tuning stage are provided in Section 4.3.

### 4.3. Mixture-Only Blind Fine-Tuning (Stage II)

Blind fine-tuning adapts a pretrained separator to unlabeled mixtures using a self-supervised objective. Given an unlabeled mixture y, the model outputs the desired estimate ${\hat{s}}_{c}$ and jammer estimate ${\hat{u}}_{c}$. These two components are recombined under the assumed nonlinear mixing model to form a reconstructed mixture ${\hat{y}}_{c}$.

(37) $${\hat{z}}_{c}[n]={\hat{s}}_{c}[n]+{\hat{u}}_{c}[n],$$

(38) $${\hat{y}}_{c}[n]={\hat{z}}_{c}[n]+a_{\mathrm{mix}}{\hat{z}}_{c}[n]|{\hat{z}}_{c}[n]{|}^{2}.$$

The parameter $a_{\mathrm{mix}}$ denotes the cubic coefficient of the reconstruction mixer used in Stage II. It controls the strength of the nonlinear correction term ${\hat{z}}_{c}[n]|{\hat{z}}_{c}[n]{|}^{2}$, and thus determines how strongly the reconstructed mixture departs from the purely linear sum in Equation (37).

Equation (37) represents the latent linear recombination of the estimated desired and jammer components, whereas Equation (38) maps this recombination into the observation domain under the assumed nonlinear front-end model. Because the received mixture in this work is generated after cubic nonlinear distortion rather than at the linear summation stage, using Equation (37) alone would impose a mismatched linear reconstruction objective. In contrast, Equation (38) enables blind fine-tuning to match the observed mixture in the same nonlinear domain in which the receiver waveform is formed. Equation (37) can therefore be viewed as an intermediate latent representation, whereas Equation (38) defines the actual reconstruction used for mixture consistency.

The reconstruction term penalizes mismatch between y and $\hat{y}$ in both the time domain and the frequency domain (log-magnitude spectra), promoting consistency with the observed mixture under the assumed nonlinearity.

(39) $$L_{\mathrm{rec},\mathrm{time}}=\frac{1}{T}∥{\hat{y}}_{c}-y_{c}{∥}_{2}^{2},$$

(40) $$L_{\mathrm{rec},\mathrm{freq}}=\frac{1}{T}\log\left(|\mathrm{FFT}({\hat{y}}_{c})|+\varepsilon \right)-\log{\left(|\mathrm{FFT}(y_{c})|+\varepsilon \right)}_{2}^{2}.$$

To discourage leakage between sources without labels, we penalize the normalized complex cross-correlation between ${\hat{s}}_{c}$ and ${\hat{u}}_{c}$, which serves as a simple surrogate measure of source disentanglement.

(41) $$L_{\mathrm{ind}}=\frac{\left|\langle {\hat{s}}_{c},{\hat{u}}_{c}\rangle \right|}{∥{\hat{s}}_{c}{∥}_{2}∥{\hat{u}}_{c}{∥}_{2}+\varepsilon }.$$

We further regularize the desired component with a constant-modulus (CM) penalty, leveraging a classical blind-equalization prior that remains effective as weak supervision for spread-spectrum signals.

(42) $$L_{\mathrm{CM}}=\frac{1}{T}\sum_{n=0}^{T-1}{\left(|{\hat{s}}_{c}[n]{|}^{2}-1\right)}^{2}.$$

This term is used only as a weak regularizer rather than a hard signal model.

Because DSSS waveforms are spectrally spread by construction, we additionally impose a spectral-flatness regularizer on the desired estimate to reduce residual narrowband structure.

(43) $$\mathrm{SF}(\hat{s})=\frac{\exp(mean(\log\left(\mathrm{PSD}(\hat{s})\right)))}{mean(\mathrm{PSD}(\hat{s}))}$$

(44) $$L_{\mathrm{flat}}=1-\mathrm{SF}(\hat{s})$$

To limit drift during unsupervised adaptation, we add a teacher-consistency term in which a frozen copy of the pretrained model (teacher) constrains the student outputs, improving stability under mixture-only updates.

(45) $$L_{\mathrm{teacher}}={\left\|\mathrm{Align}(\hat{s},{\hat{s}}_{\mathrm{teacher}})-{\hat{s}}_{\mathrm{teacher}}\right\|}_{2}^{2}$$

Here, Align(⋅,⋅) denotes a single-complex-scalar alignment operator used to remove the global complex gain/phase ambiguity before computing teacher consistency. Specifically, for two complex sequences a and b, we define

(46) $$\mathrm{Align}(a,b)=\alpha a,\;\alpha =\frac{\langle a,b\rangle }{\langle a,a\rangle +\varepsilon },$$

where $\langle \cdot ,\cdot \rangle$ denotes the Hermitian inner product. In Equation (45), this means that the teacher output is globally aligned before the $l_{2}$ penalty is evaluated, so that the consistency term is insensitive to trivial complex scale/phase differences and instead penalizes only meaningful waveform disagreement.

Finally, a weak energy-floor constraint is included to prevent trivial solutions such as ${\hat{u}}_{c}\approx 0$ that can satisfy reconstruction under certain weightings.

(47) $$P(\hat{x})≜\frac{1}{T}\sum_{n=0}^{T-1}|\hat{x}[n]{|}^{2},$$

(48) $$L_{\mathrm{floor}}=\mathrm{ReLU}\left(\rho \tau -\frac{P({\hat{u}}_{c})}{P({\hat{s}}_{c})+P({\hat{u}}_{c})+\varepsilon }\right).$$

The full blind fine-tuning objective combines reconstruction, disentanglement, and prior regularization terms with fixed weights.

(49) $$L_{\mathrm{blind}}=\beta_{t}L_{\mathrm{rec},\mathrm{time}}+\beta_{f}L_{\mathrm{rec},\mathrm{freq}}+\beta_{i}L_{\mathrm{ind}}+\beta_{cm}L_{\mathrm{CM}}+\beta_{sf}L_{\mathrm{flat}}+\beta_{T}L_{\mathrm{teacher}}+\beta_{J}L_{\mathrm{floor}}$$

All loss weights β remain fixed during blind fine-tuning. Unless otherwise stated, we fine-tune for 10 epochs using AdamW with early stopping based on reconstruction diagnostics to avoid over-adaptation.

In practice, $a_{\mathrm{mix}}$ is chosen as a moderate nominal value within the expected receiver-nonlinearity range and is used as a reconstruction-model parameter rather than as a precisely known physical constant. In our implementation, the cubic reconstruction coefficient is initialized to 0.3 and constrained by an upper bound of 1.0, which is consistent with the synthetic nonlinearity range used in the experiments.

## 5. Results and Discussion

This section presents separation results for the nonlinear, multi-observation anti-jamming scenario introduced in Section 3. We first describe the experimental design, including the labeled synthetic mixtures used for supervised pretraining and testing and the mixture-only interface that emulates deployment constraints. We then report aggregate and distributional results, followed by robustness analyses with respect to JSR, SNR, and nonlinear distortion strength. Finally, we provide ablation studies and deployment-oriented considerations.

### 5.1. Experimental Setup

This subsection summarizes the evaluation protocol and datasets. We considered two regimes: (i) labeled synthetic mixtures for supervised pretraining and controlled testing, and (ii) unlabeled mixtures for optional mixture-only adaptation, which reflects deployment conditions where clean references are unavailable.

#### 5.1.1. Synthetic Signal Model and Data Generation

We synthesized complex-baseband I/Q mixtures to enable controlled sweeps over interference strength, thermal noise, channel impairments, and front-end nonlinearity. Each example was generated in five steps. First, we synthesized a DSSS desired waveform by selecting a modulation format and spreading factor and by generating a random symbol sequence with a random ±1 spreading code. Second, we synthesized a multi-tone narrowband interferer, optionally with burst gating, and scaled its power to achieve a target jammer-to-signal ratio (JSR). Third, we applied receiver and channel impairments, including Doppler/CFO, random-walk phase noise, and sparse multipath. Fourth, we formed a nonlinear mixture using a memoryless third-order polynomial to emulate front-end compression. Finally, we added complex AWGN to reach the target SNR, defined relative to the desired component.

No separate hardware sensor-calibration procedure was performed in this study; instead, calibration-related operations refer to per-segment JSR power calibration during synthetic mixture generation, RMS normalization for mixture-only inputs, and complex-scalar alignment used in waveform-level evaluation.

Unless otherwise stated, we segmented mixtures to a fixed length T and generated them at a fixed sampling rate. Table 3 summarizes the primary operating parameters swept in our experiments (T, fs, modulation/SF, SNR, JSR, nonlinearity coefficient a, and jammer tone count M). Unless otherwise stated, receiver/channel impairments (Doppler/CFO, phase noise, and sparse multipath) are applied using fixed default settings described in Section 3.4 and kept identical across all experiments. Table 4 reports the dataset sizes and splits used for supervised pretraining and evaluation.

In the multi-observation setting, each mixture was recorded by two receivers, and learning-based models took as input the stacked real I/Q representation across receivers (four channels total). For supervised training and evaluation, desired and jammer targets were defined on a designated reference receiver ($r_{\mathrm{ref}}=0$) to ensure consistent scoring while still allowing the separator to exploit cross-receiver diversity.

We emphasize that the present synthetic evaluation is intentionally limited to the scenario family summarized in Table 3. In particular, the jammer model considered in this study is a multi-tone interferer with optional burst gating and M∈{1,…,4}. Therefore, the reported results should be interpreted as evidence of effectiveness within this bounded nonlinear anti-jamming regime, rather than as a claim of universal generalization to arbitrary deployment conditions.

The no-jamming case (M=0) is outside the current supervised training distribution and was not evaluated separately in the present manuscript. Since the separator has a fixed two-branch output structure, it would still produce a desired estimate and a jammer estimate under such an input; in the ideal case, the jammer branch should collapse to near-zero energy while the desired branch remains close to the clean received waveform, but this behavior has not been quantitatively validated here.

#### 5.1.2. Mixture-Only Dataset Interface for Blind Fine-Tuning

To emulate deployment constraints, we also considered an unlabeled regime in which only the received mixture y[n] is available. The mixture-only dataset interface returns normalized mixture segments (single- or multi-receiver recordings) without ground-truth sources, enabling self-supervised adaptation via reconstruction- and prior-based objectives.

Each retrieved segment was RMS-normalized to mitigate unknown front-end gain variations, improve numerical stability, and match the constant-modulus regularization used in the blind objective. For long recordings, we sampled random crops of length T to increase per-epoch diversity while keeping memory and compute bounded. The interface supports both single-receiver mixtures and multi-receiver mixtures stored as complex-valued arrays, which are converted to stacked real I/Q tensors before being passed to the separator.

### 5.2. Results: Overall Separation Performance

#### 5.2.1. Metrics and Evaluation Protocol

All waveform metrics were computed on the reference receiver after resolving the inherent complex scale/phase ambiguity. For evaluation, we apply the same complex-scalar alignment used during supervised training. Specifically,

(50) $$\alpha^{⋆}=\frac{\langle \hat{s},s\rangle }{\langle \hat{s},\hat{s}\rangle +\epsilon },\tilde{s}=\alpha^{⋆}\hat{s}$$

We then computed the metrics on the aligned estimate $\tilde{s}$ and the target s. The same protocol was applied to jammer estimates when available.

We report complex SI-SNR (dB) for both the desired signal and the jammer as

(51) $$\mathrm{SI}-\mathrm{SNR}(\tilde{s},s)=10\log_{10}\frac{∥proj_{s}(\tilde{s}){∥}_{2}^{2}}{∥\tilde{s}-proj_{s}(\tilde{s}){∥}_{2}^{2}+\epsilon }.$$

We also report the time-domain mean squared error (MSE) of the aligned complex sequences (implemented in real I/Q form).

We further report the normalized complex coherence, defined as the magnitude of the normalized complex correlation:

(52) $$\mathrm{coh}(\tilde{s},s)=\frac{\left|\sum_{n}\tilde{s}\left[n\right]s^{*}[n]\right|}{\sqrt{\left(\sum_{n}|\tilde{s}[n]{|}^{2})(\sum_{n}|s[n]{|}^{2}\right)}+\epsilon }$$

where $\epsilon =10^{-8}$. The coherence ranges from [0,1]; higher values indicate better agreement.

To capture rare but operationally critical failures, we complemented mean performance with distributional diagnostics. We report (i) a failure rate defined as SI-SNR < 0 dB and (ii) the lower-tail percentile P10 (10th-percentile SI-SNR) to summarize long-tail behavior.

We note that the present study evaluates the proposed method primarily as a receiver-side front-end separator rather than as a complete end-to-end communication receiver. For this reason, we focus on waveform-level and reliability-oriented metrics, including SI-SNR, MSE, coherence, failure rate, and lower-tail behavior, which directly quantify the fidelity and robustness of the recovered desired baseband waveform while isolating the contribution of the separator itself. A full BER evaluation would additionally require a specified downstream chain including synchronization, despreading, symbol decision, and possibly channel decoding, which is beyond the scope of the present paper.

For stratified diagnostics on synthetic data (where ground truth is available), we estimated nuisance variables on the reference receiver and used them only for binning and trend plots. JSR was computed from component powers. An effective cubic coefficient â was obtained by least squares from $y\approx z+az|z{|}^{2}$, where $z=s_{c}+u_{c}$, and an effective SNR was computed from the residual after reconstructing y using $\hat{a}$. These estimates were used solely for stratified evaluation and did not affect training.

#### 5.2.2. Baseline Methods and Comparison Settings

We compared the proposed approach with four baselines spanning (i) no separation, (ii) classical narrowband interference (NBI) mitigation prior to despreading, (iii) nonlinear blind source separation, and (iv) a strong supervised deep separator. All methods were evaluated on the same reference receiver ($r_{\mathrm{ref}}=0$) using the metric protocol in Section 5.2.1. The no-separation baseline and the Multi-Notch + Blanking baseline operate on the reference-channel mixture only, reflecting conventional single-channel front-end mitigation, whereas Kernel-FastICA and Conv-TasNet use the stacked dual-receiver real I/Q input.

As a no-separation baseline, we used the reference-channel mixture $y_{m}$ directly as the desired estimate ($\hat{s}=y_{m}$).

For the Multi-Notch + Blanking baseline, we implemented a conventional narrowband interference mitigation front end on the reference-channel mixture $y_{m}$. The method first notches the K strongest spectral lines and then applies time-domain blanking to high-energy samples to suppress burst interference. Unless stated otherwise, the mitigated output is used as the desired estimate $\hat{s}$, with $\hat{J}=y_{m}-\hat{s}$ used only for evaluation consistency. The hyperparameters were fixed to K=4, Q=200, τ=8dB, and W=32 samples, all selected exclusively on the validation set.

For the Kernel-FastICA baseline, we applied Kernel-FastICA to the stacked real I/Q observations from the two receivers. Two components were extracted using an RBF kernel with a fixed bandwidth of σ=1.0, max=300, and $tol=1\times 10^{-6}$. All hyperparameters were selected on the validation set only. Because ICA is permutation- and scale-ambiguous, the output ordering was fixed using a validation-determined rule before evaluation on the test set.

As a strong supervised deep separation baseline, we implemented a time-domain encoder–separator–decoder architecture following Conv-TasNet. The network takes the stacked four-channel real I/Q input and outputs two separated sources (desired and jammer) on the reference receiver. It was trained on the same labeled synthetic dataset with the same train/validation split and training budget as the proposed model, using permutation-invariant training (PIT). In our experiments, Conv-TasNet is used as a representative strong state-of-the-art deep separation baseline under the same input/output setting and training budget. Metrics were computed using the protocol in Section 5.2.1.

To keep the comparison as consistent as possible, all baseline hyperparameters were tuned on the held-out validation set only, and the test set was used once for final reporting. All learning-based methods shared identical data splits and were evaluated with the same alignment procedure and metric implementation, while conventional single-channel baselines were included to represent standard front-end mitigation practice.

#### 5.2.3. Overall Performance and Distributional Diagnostics

**Figure 5:**
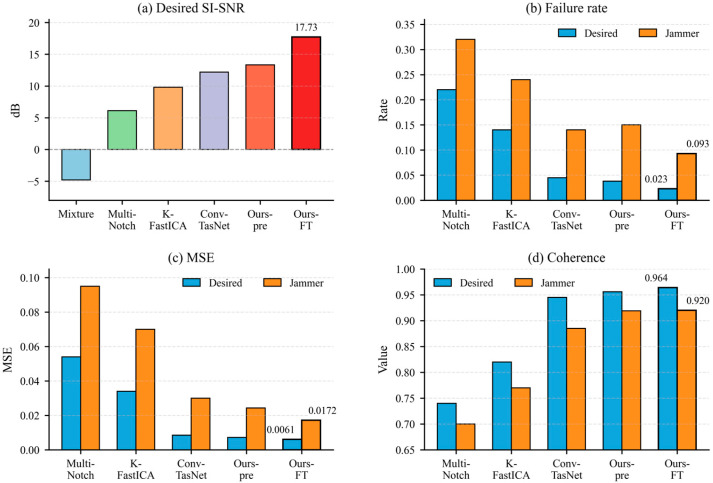
Overall performance comparison on the synthetic test set. Ours-pre denotes Stage I supervised pretraining only, and Ours-FT denotes Stage I pretraining followed by Stage II blind fine-tuning.

We evaluated all methods on a held-out synthetic test set generated according to Table 3 (N=1000 mixtures). Figure 5 compares overall performance across methods in terms of desired-signal SI-SNR, failure rate, MSE, and coherence. Relative to the mixture-as-estimate baseline, supervised pretraining improved the desired-signal SI-SNR from −4.79 ± 14.35 dB to 13.32 ± 8.73 dB. Mixture-only blind fine-tuning further increased the SI-SNR to 17.73 ± 8.65 dB and reduced the desired-signal failure rate from 3.8% to 2.3%. For the jammer estimate, SI-SNR increased from 13.23 ± 9.50 dB to 16.21 ± 11.09 dB, and the jammer failure rate dropped from 15.0% to 9.3%.

These results can be understood as follows: The proposed method outperforms the conventional and nonlinear baselines mainly because it combines complementary inductive biases in both the architecture and the training strategy. The multi-scale convolutional front end captures local chip-level DSSS structure together with narrowband spectral patterns of the jammer, whereas the Transformer encoder models longer-range temporal dependencies associated with burst activity and with time-varying impairments such as Doppler/CFO and phase noise. In addition, Stage II mixture-only fine-tuning improves performance under domain shift by enforcing reconstruction consistency in the nonlinear observation domain and by suppressing source leakage through weak communication-motivated priors. By contrast, performance still degrades as JSR increases, since stronger interference makes separation more ambiguous and raises the risk of rare catastrophic failures; moreover, blind adaptation must be applied conservatively when the assumed reconstruction model is mismatched to the target hardware.

Overall, these results provide a consistent comparison with conventional, nonlinear BSS and deep-learning baselines and show that the proposed method delivers the strongest overall performance under a common evaluation protocol.

Because mean SI-SNR can be sensitive to outliers and may obscure rare but operationally important failures, we also report reliability-oriented distributional diagnostics, particularly the failure rate and lower-tail behavior. These quantities are further examined in the ablation analysis.

In deployment, mixture-only fine-tuning is intended for domain-shift scenarios in which labeled references are unavailable and mixture statistics differ from those seen during pretraining. Conservative objective weighting and early stopping are important to avoid over-adaptation when the assumed reconstruction model does not fully match the target hardware. Reconstruction agreement (y versus $\hat{y}$) and leakage indicators (e.g., coherence between ${\hat{s}}_{c}$ and ${\hat{u}}_{c}$) provide lightweight diagnostics for monitoring adaptation stability.

### 5.3. Robustness Analysis

**Figure 6:**
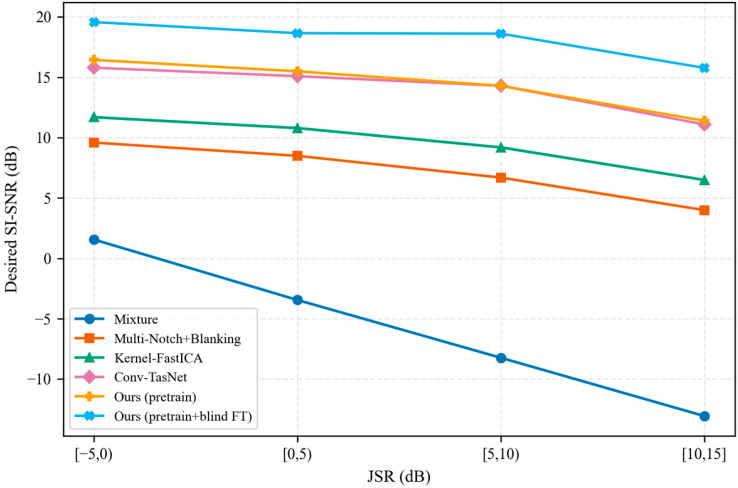
Desired-signal separation performance across different jammer-to-signal-ratio (JSR) levels. Results are reported as average SI-SNRs within 5 dB JSR bins. FT denotes blind fine-tuning.

To assess robustness with respect to JSR under interference-dominant conditions, we stratified the test set into 5 dB JSR bins and report binned statistics. Figure 6 shows the desired-component SI-SNR trends across JSR bins for all methods.

At high JSR, average performance can mask rare catastrophic collapses that are critical in protected-link operation. Accordingly, we emphasize reliability-oriented diagnostics (tail percentiles and failure rates) in addition to binned means. Across the evaluated JSR range, the proposed model degraded more gracefully than the mixture baseline, and mixture-only fine-tuning provided the largest benefits in the high-JSR regime.

### 5.4. Ablation and Practical Considerations

Mixture-only adaptation targets deployment scenarios where clean references are unavailable and mixture statistics differ from those used in supervised pretraining. Under such conditions, self-supervised fine-tuning can improve robustness, provided that objective weights and stopping criteria are chosen conservatively.

When labeled data are available (e.g., in simulation), we quantify the effect of adaptation by comparing SI-SNR before and after mixture-only fine-tuning. In fully unsupervised deployment, reconstruction agreement (y versus $\hat{y}$) and leakage indicators (e.g., $\hat{s}-\hat{j}$ coherence) provide practical proxies for monitoring adaptation stability.

From an architectural perspective, the separator combines a multi-scale convolutional front end with a Transformer encoder. The parallel temporal convolutions capture local chip-level structure of DSSS as well as narrowband spectral patterns typical of multi-tone jammers, while the Transformer aggregates longer-range context to track burst activity and time-varying impairments such as Doppler/CFO and phase noise. Long-context modeling is particularly relevant in spread-spectrum reception: the desired signal exhibits structured repetition over chip and symbol intervals, whereas the jammer may vary abruptly in time. Self-attention can exploit these differences to stabilize separation when instantaneous SNR and JSR are unfavorable.

#### 5.4.1. Ablation: Effect of Blind Fine-Tuning

**Figure 7:**
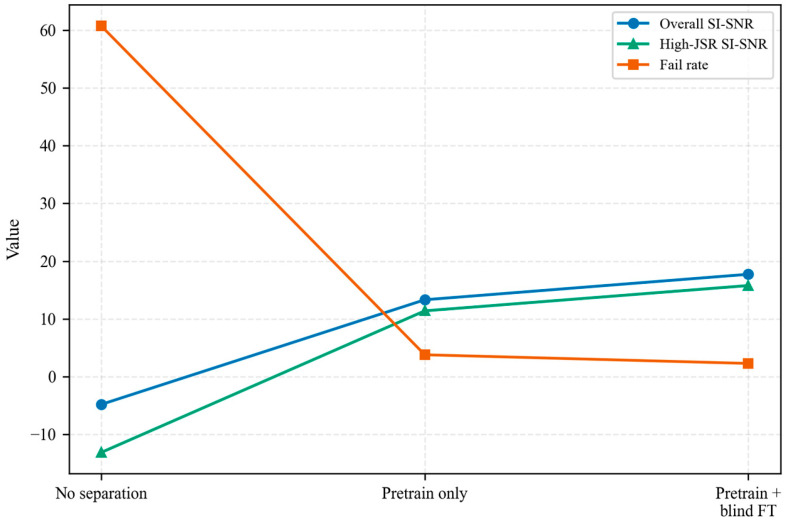
Effect of blind fine-tuning on separation performance. The figure compares no separation, Stage I pretraining only, and Stage I followed by Stage II blind fine-tuning. High-JSR refers to the subset with JSR ≥ 10 dB.

To quantify the effect of mixture-only fine-tuning, Figure 7 compares the pretrained model with its mixture-only fine-tuned counterpart and also reports results for the high-JSR subset. This additional subset analysis highlights behavior in the worst-case interference regime.

**Figure 8:**
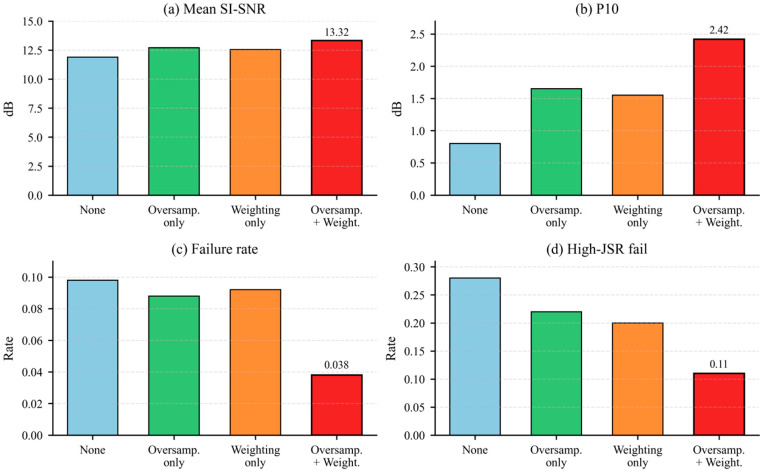
Ablation study of high-JSR oversampling and JSR-aware loss weighting during Stage I supervised pretraining.

Complementing the adaptation ablation in Figure 7, Figure 8 presents a targeted 2 × 2 study of two Stage I robustness mechanisms: high-JSR oversampling and JSR-aware loss weighting. Further ablations within the blind objective (e.g., removing teacher consistency or individual priors) are left for future work.

The observed gain from blind fine-tuning indicates that Stage II is not a redundant repetition of Stage I, but an adaptation step that uses unlabeled target mixtures to reduce residual domain mismatch after supervised synthetic pretraining.

#### 5.4.2. Ablation: High-JSR Oversampling and JSR-Aware Loss Weighting

To isolate the contribution of the two robustness mechanisms introduced in Stage I, Figure 8 presents a targeted 2 × 2 ablation restricted to supervised pretraining: high-JSR oversampling and JSR-aware weighting of the desired-signal loss. The evaluated configurations are none, oversampling only, weighting only, and both combined. All other settings, including the architecture, optimizer, training budget, and test protocol, were kept fixed.

Figure 8 illustrates the effect of high-JSR oversampling and JSR-aware loss weighting on mean SI-SNR, P10, failure rate, and high-JSR failure rate. Across the evaluated configurations, these mechanisms primarily improved reliability (tail percentile P10 and failure rate), with the largest benefits concentrated in the high-JSR subset (JSR ≥ 10 dB). This supports the use of reliability-oriented objectives and sampling when targeting anti-jamming receivers that are sensitive to rare failure events.

#### 5.4.3. Computational Complexity

We summarize the model size and the asymptotic computational cost as functions of sequence length T, hidden dimension d, and encoder depth L. For a standard Transformer encoder, multi-head self-attention dominates runtime with $O({\mathrm{LT}}^{2}d)$ complexity and $O(T^{2})$ attention-memory usage, whereas the convolutional front end contributes O(Td) overhead. In practice, the multi-scale convolutions add modest linear-time cost while improving local feature extraction, and overall complexity is primarily governed by the attention layers at the chosen T.

Because the current Transformer backbone has quadratic attention complexity with respect to sequence length, the present implementation is not yet specifically optimized for highly resource-constrained edge devices; such deployment would likely require efficient-attention, compression, quantization, or hardware-accelerated inference.

#### 5.4.4. Nuisance-Parameter Estimation

When ground truth is available (synthetic evaluation), we estimate JSR, SNR, and the effective nonlinearity coefficient using the component-based procedures described in Section 5.2.1. These quantities are used only for stratified analysis (binning and trend plots) and are not required by the separator during inference.

### 5.5. Deployment Considerations, Limitations, and Future Directions

Overall, the results suggest that the separator can serve as a front-end module before conventional synchronization, despreading, and detection, thereby complementing classical anti-jamming processing. The largest gains occurred in high-JSR regimes where baseline processing is prone to catastrophic failures, which is consistent with protected-link availability requirements.

In modern satellite communication systems, this separator is not intended to replace beamforming, multibeam reception, or higher-layer resource management. Instead, it can be incorporated as a receiver-side nonlinear interference mitigation module within each beam, terminal, or gateway receive chain, complementing spatial filtering and subsequent baseband processing. This interpretation is particularly relevant to broadband satellite systems in which advanced receiver architectures coexist with strong interference and possible front-end nonlinearity.

From a practical deployment perspective, the method assumes the required observation channels and digital baseband inference resources, but it does not alter the surrounding beamforming or higher-layer resource-management architecture. Its scalability is modular because the separator can be instantiated independently for each beam, terminal, or gateway receive chain, while the per-instance implementation cost is dominated primarily by the computational and memory complexity discussed in Section 5.4.3.

Several limitations bound the current conclusions. First, the jammer model focuses on multi-tone interference with optional burst gating; operational jammers can be swept, modulated, reactive, or protocol-aware. Second, while the channel model includes Doppler/CFO, phase noise, and sparse multipath, it does not capture all propagation effects. Third, the nonlinearity model is a memoryless cubic polynomial; real front-end nonlinearities may exhibit memory and frequency selectivity that require richer models. Finally, while SI-SNR and coherence quantify waveform fidelity and are appropriate for isolating front-end separation performance, link-level metrics such as post-despreading processing gain and bit-error rate would provide a more system-oriented evaluation. Such BER evaluation requires an explicit downstream receiver chain, including synchronization, despreading, symbol decision, and possibly decoding, which is beyond the scope of the present front-end separation study and is therefore left for future work.

More generally, when the received signal is generated outside the ranges or jammer types covered by Table 3, the separator will still output two waveforms because of its fixed desired/jammer decomposition, but the separation quality is not guaranteed by the present experiments. In practice, inputs that remain close to the training distribution may be handled with graceful degradation, whereas substantial mismatch may reduce reliability. The optional mixture-only blind fine-tuning stage is intended to mitigate such moderate simulation-to-deployment mismatch, but it should not be interpreted as a guarantee of robustness under arbitrary out-of-distribution conditions.

Future work will validate the approach on real receiver recordings and under deliberately mismatched simulation conditions, broaden jammer diversity, and extend the distortion model (e.g., learnable memory-polynomial mixers). Another direction is to integrate differentiable despreading/decoding modules so that task-level objectives can be optimized end-to-end for task-level metrics (e.g., post-despreading BER). Another important future direction is to replace the current stacked real-valued I/Q implementation with a native complex-valued separator, such as a complex-valued Transformer, so as to more fully exploit the algebraic coupling between the real and imaginary components of communication signals. Recent score-based RF source-separation models further suggest that learned generative priors may be a promising direction for future communication-signal separation systems [31].

## 6. Conclusions

This study investigated anti-jamming reception for direct-sequence spread-spectrum (DSSS) satellite links under strong multi-tone or burst interference and receiver front-end nonlinearity. By formulating the task as nonlinear source separation in complex baseband, we developed a time-domain Transformer-based separator that operates on stacked real-valued I/Q tensors derived from complex-baseband observations and jointly estimates the desired DSSS signal and the jammer.

To improve reliability under severe interference, we adopted a two-stage training strategy. Stage I uses supervised pretraining on synthetically generated nonlinear mixtures with high-JSR oversampling and JSR-aware loss weighting, whereas the optional Stage II performs mixture-only blind fine-tuning based on nonlinear reconstruction consistency and communication-motivated priors. On the synthetic test set (N=1000; JSR∈[−5,15] dB; SNR∈[15,25] dB; a∈[0,0.5]), supervised pretraining increased the desired-component SI-SNR from −4.79 ± 14.35 dB for the mixture baseline to 13.32 ± 8.73 dB and reduced the failure rate (SI-SNR < 0 dB) from 60.7% to 3.8%. Mixture-only blind fine-tuning further improved the desired-component SI-SNR to 17.73 ± 8.65 dB and reduced the failure rate to 2.3%. The gains were especially pronounced in the high-JSR regime (10–15 dB), where the baseline degraded markedly, the pretrained model maintained robust separation, and blind fine-tuning provided an additional improvement within that bin (Figure 6).

Overall, these results indicate that the proposed separator is an effective nonlinear anti-jamming front end for DSSS reception in the evaluated setting. Although mixture-only blind fine-tuning was assessed here in a controlled synthetic regime, its consistent gains beyond supervised pretraining indicate that reconstruction-consistency objectives and communication-motivated priors can provide practical additional robustness when clean references are unavailable. Future work will extend the evaluation to real receiver recordings, broader jammer models, richer nonlinear front-end representations, and link-level metrics such as post-despreading processing gain and bit-error rate.

## Figures and Tables

**Table 1:** Main architectural hyperparameters.

Component	Setting
Encoder layers	6
Model dimension	256
Attention heads	8
Feed-forward dimension	512
Front-end temporal kernels	3, 7, 15
Output sources	2 (desired signal and jammer)

**Table 2:** Key settings and default loss weights for supervised pretraining.

Item	Setting
Input	Dual-receiver I/Q input; sequence length 1024
Backbone	Transformer-based separator (6 encoder layers, model dimension 256, 8 attention heads)
Optimization	AdamW optimizer; batch size 32; 30 epochs
Time-domain weight	$\lambda_{t}=1.5$
Frequency-domain weight	$\lambda_{f}=0.5$
Complex-coherence weight	$\lambda_{c}=0.3$
SI-SNR weight	$\lambda_{\mathrm{si}}=2.0$
Scale-anchor weight	$\lambda_{\mathrm{sc}}=0.02$
Jamming supervision	Enabled with auxiliary weight 0.1
High-JSR oversampling	Enabled during training
Oversampling policy	Mixture distribution: [−5,10] dB and [10,15] dB, with high-JSR sampling probability 0.60
JSR-aware loss weighting	Enabled for the desired-signal loss only
JSR-weighting policy	Activated above 5 dB JSR; linear slope 0.20; capped multiplier 3.0; normalized to mean 1 per batch

**Table 3:** Default synthesis parameter settings used in the experiments.

Category	Setting
Sequence length	1024 samples
Sampling rate	10 MHz
DSSS modulation	BPSK, QPSK
Spreading factor	16, 32, 64
SNR range	15 to 25 dB
JSR range	−5 to 15 dB
Nonlinearity coefficient	0 to 0.5
Number of jammer tones	1 to 4

**Table 4:** Synthetic dataset configuration used in supervised training and evaluation.

Item	Setting
Dataset split (train/val/test)	8000/1000/1000
Number of receivers	2
Reference receiver	Receiver 0
Supervision targets	Desired signal and jammer on the reference receiver
Training-time JSR emphasis	High-JSR samples oversampled during supervised pretraining

## Data Availability

The data presented in this study are available on request from the first author.

## Abbreviations

The following abbreviations are used in this manuscript:

AWGN	Additive white Gaussian noise
BSS	Blind source separation
CFO	Carrier-frequency offset
CMA	Constant-modulus algorithm
DSSS	Direct-sequence spread spectrum
EMA	Exponential moving average
GNSS	Global Navigation Satellite System
ICA	Independent component analysis
IoT	Internet of Things
JSR	Jammer-to-signal ratio
PIT	Permutation-invariant training
SI-SNR	Scale-invariant signal-to-noise ratio
SNR	Signal-to-noise ratio
STAP	Space–time adaptive processing
TDL	Tapped-delay line
